# STRUCTURAL INFLUENCES ON WHY OLDER ASIAN IMMIGRANTS PROVIDE CARE FOR SPOUSES WITH DEMENTIA

**DOI:** 10.1093/geroni/igae098.3383

**Published:** 2024-12-31

**Authors:** Julie Kim, Jung-Ah Lee

**Affiliations:** University of California Irvine, Irvine, California, United States; University of California Irvine, Irvine, California, United States

## Abstract

Elder care and dementia caregiving studies on Asian immigrant families commonly cite cultural expectations, filial piety, and Confucianism as motivations for substantial reliance on informal caregivers such as adult children and children-in-law. Yet in Asian immigrant families of older adults with dementia, spouses assume primary caregiving roles as much as daughters and daughters-in-law do. This study moves beyond cultural arguments by exploring the influence of structural forces that leave caregiving responsibilities to spouses rather than adult children, or other caregivers, in Asian immigrant families of older adults with dementia. Data come from a larger dementia caregiver home-visit-based intervention study with bilingual and bicultural community health workers (CHW) visiting caregivers. The present study employs content analysis of CHW’s home visit logs with 14 Korean immigrant (10 wives) spousal caregivers and 18 Vietnamese immigrant (10 wives) spousal caregivers, both ethnic groups associated with strong cultural norms of filial piety. Detailed home visit logs contain observations of living arrangements and environments and recount conversations and events from each home visit, contextualizing the social and physical environments of caregivers and spouses with dementia. Findings show Asian immigrant spousal caregivers do not expect high levels of care support from their adult children, as assumed in cultural arguments. Instead, structural influences, such as ethnic enclaves and resources therein, the couples’ relative socioeconomic positions, access to institutional resources, and changing structures in family relationships, shape spousal caregiving arrangements and modify older immigrants’ expectations of adult children.

